# The reliability of the twelve-item general health questionnaire (GHQ-12) under realistic assumptions

**DOI:** 10.1186/1471-2458-8-355

**Published:** 2008-10-14

**Authors:** Matthew Hankins

**Affiliations:** 1 King's College London, Department of Psychology (at Guy's), Institute of Psychiatry, London, UK; 2 Department of Primary Care & Public Health, Brighton & Sussex Medical School, Brighton, UK; 3 Brighton & Sussex University Hospitals NHS Trust, Royal Sussex County Hospital, Brighton, UK

## Abstract

**Background:**

The twelve-item General Health Questionnaire (GHQ-12) was developed to screen for non-specific psychiatric morbidity. It has been widely validated and found to be reliable. These validation studies have assumed that the GHQ-12 is one-dimensional and free of response bias, but recent evidence suggests that neither of these assumptions may be correct, threatening its utility as a screening instrument. Further uncertainty arises because of the multiplicity of scoring methods of the GHQ-12. This study set out to establish the best fitting model for the GHQ-12 for three scoring methods (Likert, GHQ and C-GHQ) and to calculate the degree of measurement error under these more realistic assumptions.

**Methods:**

GHQ-12 data were obtained from the Health Survey for England 2004 cohort (n = 3705). Structural equation modelling was used to assess the fit of [1] the one-dimensional model [2] the current 'best fit' three-dimensional model and [3] a one-dimensional model with response bias. Three different scoring methods were assessed for each model. The best fitting model was assessed for reliability, standard error of measurement and discrimination.

**Results:**

The best fitting model was one-dimensional with response bias on the negatively phrased items, suggesting that previous GHQ-12 factor structures were artifacts of the analysis method. The reliability of this model was over-estimated by Cronbach's Alpha for all scoring methods: 0.90 (Likert method), 0.90 (GHQ method) and 0.75 (C-GHQ). More realistic estimates of reliability were 0.73, 0.87 and 0.53 (C-GHQ), respectively. Discrimination (Delta) also varied according to scoring method: 0.94 (Likert method), 0.63 (GHQ method) and 0.97 (C-GHQ method).

**Conclusion:**

Conventional psychometric assessments using factor analysis and reliability estimates have obscured substantial measurement error in the GHQ-12 due to response bias on the negative items, which limits its utility as a screening instrument for psychiatric morbidity.

## Background

The twelve-item General Health Questionnaire (GHQ-12) is intended to screen for general (non-psychotic) psychiatric morbidity [1]. It has been widely used and, as a result, translated into many languages and extensively validated in general and clinical populations worldwide [2]. The validation process has been principally psychometric in nature, focusing on the reliability and validity of the data generated, with additional support coming from studies of the sensitivity and specificity of the measurement [2,3]. Despite this, the utility of using self-report measures such as the GHQ-12 has been questioned, with a recent review concluding that clinicians may find the low positive predictive value of this method unconvincing as a diagnostic aid [4]. This raises the question of whether psychometric validation alone is a sufficient basis for adopting the GHQ-12 as a screening instrument in clinical practice. In clinical practice, poor positive predictive value means that many of those screening positive are not suffering from a psychiatric disorder but may be deemed to warrant further investigation; in a research context it means that many participants will be misclassified, a form of measurement error that will bias subsequent analyses [5].

In classical test theory, a test or questionnaire is assessed for dimensionality, reliability and validity [6]. Dimensionality is assessed using factor analysis, a method based on the pattern of correlations between the questionnaire item scores. If all items share moderate to strong correlations, this produces a single 'factor' and suggests that the scale measures a single dimension. Several groups of such items produce several factors, suggesting that several dimensions are being measured. Since the method depends on the inter-item correlations, anything that produces correlated items will be interpreted as a factor, and therefore caution should be exercised when interpreting factor structures as substantive dimensions [6]. Reliability is an estimate of the degree of measurement error entailed in the measurement of a single dimension by several items. If a questionnaire measures several dimensions, then each requires an estimate of reliability. Several methods are commonly used to estimate reliability (for example, Cronbach's Alpha or test-retest correlations), but all rely on the correlation between items (Alpha) or scale scores (test-retest). In addition, the interpretation of the resulting reliability coefficient depends on some strong assumptions being met: most notably in the context of the current study, there is the assumption that the measurement error of each item is random (i.e. uncorrelated with anything else). Finally, validity refers to the extent to which the test or questionnaire measures what it is supposed to measure. This is commonly assessed with reference to some external criterion, but it should be clear that a questionnaire intended to measure a single dimension cannot be valid if it measures several dimensions, or if it produces data with a high proportion of measurement error. Hence, factor analysis and reliability estimates contribute to the sufficiency of a measure, but do not guarantee it.

While psychometric evaluation of the GHQ-12 suggests that it is a valid measure of psychiatric morbidity (i.e. it measures what it purports to measure), and also a reliable measure (i.e. measurement error is low), examination of the factor structure has repeatedly led to the conclusion that the GHQ-12 measures psychiatric morbidity in more than one domain [7]. These results have been interpreted as evidence that the GHQ-12 measures more than one dimension of psychiatric morbidity, although typically each dimension has been found to be reliable and the measurement error for each dimension acceptable. Currently the consensus appears to be that the GHQ-12 measures psychiatric dysfunction in three domains, *social dysfunction*, *anxiety *and *loss of confidence *[7-9], although having been derived solely from factor analysis, both the utility and the clinical ontology of these domains remains unclear [10].

Another interpretation of this factor analytic evidence is that the apparent multidimensional nature of the GHQ-12 is simply an artefact of the method of analysis, rather than an aspect of the GHQ-12 itself [10]. The studies reporting that the GHQ-12 is multidimensional used either exploratory factor analysis (EFA) or confirmatory factor analysis by structural equation modelling (SEM), and it has long been known that these methods can produce spurious dimensions even when the measure in question is one-dimensional if the questionnaire comprises a mixture of positively phrased items and negatively phrased items [11-14]. For example, the Rosenberg Self-Esteem Scale was thought to be multidimensional on the basis of repeated factor analyses [15], but analysis of method effects [14] revealed that the 'factors' split the scale into positively and negatively phrased items, and that the data were more consistent with a one-dimensional measure with response bias on the negatively phrased items. In addition, substitution of the negatively phrased items with the same concepts expressed in positive phrases resulted in a one dimensional structure [16]. Similarly, the seemingly two-dimensional Consideration of Future Consequences Scale (CFC) [17] was found to one dimensional when response bias on the reverse-worded items was taken into account [18].

The dimensions identified for the GHQ-12 essentially split the questionnaire into positively and negatively phrased items and analysis of method effects in a large general population sample has confirmed that the data are more consistent with a one dimensional measure, albeit with substantial response bias on the negatively phrased items [10]. The response bias so identified has been attributed to the ambiguous wording of the responses to the negatively phrased items [10], where the response choices to statements such as 'Felt constantly under strain' are: 'No more than usual', 'Not at all', 'Rather more than usual' and 'Much more than usual'. The first two options apply equally well to respondents wishing to indicate the *absence *of a negative mood state. This explanation, however, depends crucially on the scoring system applied to the GHQ-12. The GHQ-12 has two recommended scoring methods: a four point response scale ('Likert method') or a two point response scale ('GHQ method'), and this ambiguity can only apply to the former; for the latter, both responses are collapsed into the same category of response (absent) and the distinction vanishes. In addition, a further scoring method ('C-GHQ' method) was devised expressly to eliminate the ambiguity of responses to the negatively phrased items [18], following the observation that someone indicating that they 'Felt constantly under strain', 'No more than usual', was probably indicating the *presence *of this negative mood state. Variation in scoring method has been found to affect the sensitivity [18], discrimination [19] and the apparent dimensionality of the GHQ-12 [7]. It may also, as argued above, affect the degree of response bias and possibly eliminate it altogether.

In summary, the poor predictive value of the GHQ-12 may be due to the multidimensional nature of the questionnaire or to response bias on the negatively phrased items: these are competing hypotheses, since the response bias is also responsible for the appearance of multidimensionality, and the multidimensional models in turn assume that there is no response bias. If the GHQ-12 is multidimensional then it will perform poorly as a screen for non-specific psychiatric morbidity; if it has a substantial degree of response bias then the problem is exacerbated because conventional indices of reliability such as Cronbach's Alpha [21] may underestimate the degree of measurement error [22,23]. Only two studies [7,10] have approached this problem in a systematic way. The first of these [7] assessed the relative fit of several competing one-, two- and three-dimensional models using the three different scoring methods, but did not model response bias. The second [10] assessed the fit of competing dimensional models, including one with response bias, but did not examine the effects of scoring method. This study therefore aimed to evaluate the GHQ-12 in terms of the three scoring methods applied to three models: the original one-dimensional model, the 'best' three-dimensional model, and a one-dimensional model incorporating response bias. Having determined the best model for the data, the second aim was to estimate the reliability of the GHQ-12 under the more realistic assumptions entailed by the model.

## Methods

### Data

GHQ-12 data were obtained from the 2004 cohort of the Health Survey for England, a longitudinal general population conducted in the UK. Sampling and methodological details are in the public domain [24]. For the purposes of this study, a single adult was selected from each household to maintain independence of data. Of the 4000 such adults, 3705 provided complete data for the GHQ-12.

### GHQ-12 coding

The GHQ-12 comprises 12 items describing mood states, six of which are positively phrased (PP items, labelled items p1 to p6) and six negatively phrased (NP items, labelled n1 to n6). Each item of the GHQ-12 has four possible response options. Item scores were coded according to the three scoring methods examined: Likert method (all items coded 0-1-2-3), GHQ method (all items coded 0-0-1-1), and C-GHQ method (PP items coded 0-0-1-1; NP items coded 0-1-1-1). Three severity scores were computed as the summed score of all items for each scoring method.

### Analysis

Structural equation modelling (SEM) using maximum likelihood estimation [26] was used to compare the three models under consideration for each scoring method. SEM is a statistical method employing factor analysis and linear regression techniques to assess the 'fit' of a model to the data, i.e. the extent to which the model is an adequate description of the data. This confirmatory approach has three advantages over conventional factor analysis. First, it allows a priori hypotheses to be tested, unlike factor analysis which is essentially a descriptive method. Second, it allows the comparison of competing models. This is particularly helpful when more than one model is an adequate fit for the data. Third, models may be specified with observed variables and unobserved (latent) variables, and associations between variables defined explicitly.

The models tested in this study were essentially measurement models, in that the interest was in how the observed variables (the item scores) were related to the latent variables (the GHQ-12 dimensions). In keeping with classical test theory, each model specified that each item was determined by one latent variable plus a unique error term.

### Model specifications

**Figure 1:**
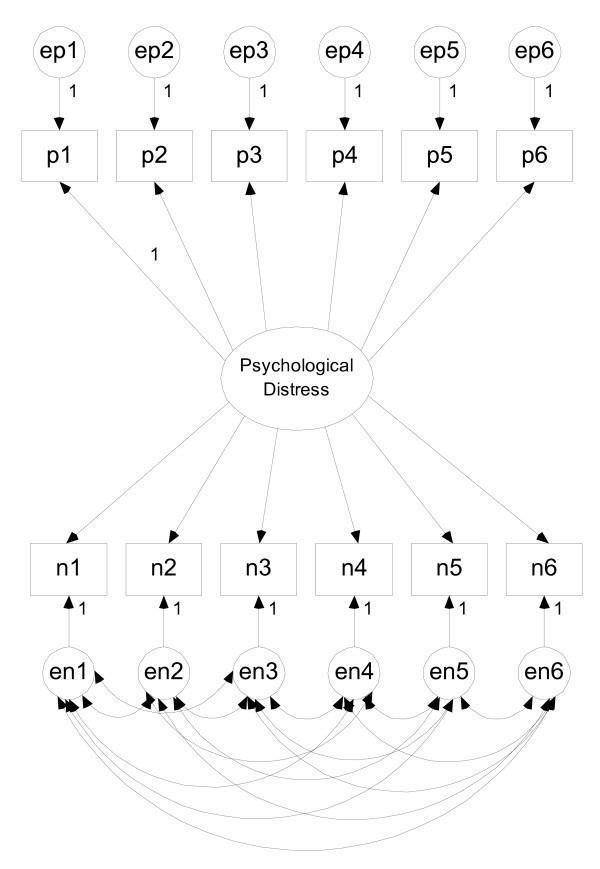
One dimension ("Psychological Distress") with correlated error terms on the negatively-phrased items.

Three models were compared for each scoring method (a total of nine models):

1. One-dimensional: the GHQ-12 was modelled as a measure of one construct (severity of psychiatric disturbance) using all 12 items, i.e. one latent variable with twelve indicator variables (items), each with its own error term;

2. Three-dimensional: the GHQ-12 was modelled as a measure of three correlated dimensions of psychiatric disturbance: *social dysfunction *(items p1 to p6), *anxiety *(items n1 to n4), and *loss of confidence *(items n5 and n6). This model was originally derived by Graetz [9] and is the best supported factorial model of the GHQ-12 [6,7]. The model specified was therefore three latent variables, each correlated with the other, with indicator variables as noted, each with its own error term;

3. One-dimensional with correlated errors: the GHQ-12 was modelled as a measure of one construct (see Figure 1) but with correlated error terms on the NP items, modelling response bias. The model specified was therefore identical to model 1, but with correlations specified between the error terms on the NP items.

### Fit indices

A range of fit indices was computed to allow for the comparison of models [26]. These indices differ in (a) how the sample covariance matrix is compared to a baseline 'null' model and (b) the extent to which sample size and model parsimony are taken into account. The normed fit index (NFI) and comparative fit index (CFI) increase towards a maximum value of 1.00 for a perfect fit, with values around 0.950 indicating a good fit for the data. The root mean square error of approximation (RMSEA), Akaike information criterion (AIC), consistent AIC (CAIC), Bayesian information criterion (BIC) and expected cross-validation index (ECVI) decrease with increasingly good fit. While the AIC, CAIC, BIC and ECVI all indicate how well the model will cross-validate to another sample, the RMSEA provides a 'rule of thumb' cutoff for model adequacy of < 0.08.

### Scale properties

The reliability of the best fitting model was estimated using Cronbach's Alpha [21] and the square of the implied correlation between the latent variables and their composite scores, using unweighted least squares regression [22,23]. From these the standard error of measurement (SEM) was calculated [6]. Scale discriminations were computed using Ferguson's Delta [19]. This index expresses the degree to which the scale discriminates between individuals and ranges from 0 to 1. A Delta of 0 indicates that no discriminations at all were made, and all respondents obtained the same score; a value of 1 indicates that all scores were made with equal frequency [19]. Delta may be interpreted as the ratio of observed discriminations to the maximum possible number; Delta = 0.80 indicates that 80% of all possible discriminations for the sample were actually made. Further examples are discussed in Hankins (2008) [26]

## Results

Table 1 shows the fit indices for all models and scoring methods.

**Table 1:** Fit indices for all models and scoring methods

**Model**	**H_0 _χ^2 (DF)^**	**χ^2 (DF)^**	**NFI**	**CFI**	**RMSEA (90% CLs)**	**BIC**	**AIC**	**CAIC**	**ECVI**
***Likert method: all items (0-1-2-3)***									
1. One dimension	20520.61 (66)	3161.52 (54)	0.85	0.85	0.13 (0.12, 0.13)	3358.74	3209.52	3382.74	0.87
2. Three dimensions	20520.61 (66)	1061.74 (51)	0.95	0.95	0.07 (0.07, 0.07)	1283.61	1115.74	1310.61	0.30
3. Response bias	20520.61 (66)	713.26 (39)	0.97	0.97	0.07 (0.06, 0.07)	1033.74	791.26	1072.74	0.21
***GHQ method: all items (0-0-1-1)***									
1. One dimension	19497.47 (66)	1487.90 (54)	0.92	0.93	0.09 (0.08, 0.09)	1685.12	1535.90	1709.12	0.42
2. Three dimensions	19497.47 (66)	1162.49 (51)	0.94	0.94	0.08 (0.07, 0.08)	1384.36	1216.49	1411.36	0.33
3. Response bias	19497.47 (66)	869.10 (39)	0.96	0.96	0.08 (0.07,0.08)	1189.58	947.10	1228.58	0.26
***C-GHQ method: PP items (0-0-1-1), NP items (0-1-1-1)***									
1. One dimension	11954.89 (66)	4006.40 (54)	0.68	0.68	0.16 (0.15, 0.16)	4203.62	5102.66	5275.88	1.38
2. Three dimensions	11954.89 (66)	916.14 (51)	0.95	0.95	0.07 (0.06, 0.07)	1138.01	917.40	1112.27	0.25
3. Response bias	11954.89 (66)	569.00 (39)	0.97	0.97	0.05 (0.05,0.06)	889.48	524.84	806.33	0.14

### Model 1

Consistent with previous findings, the simple one-dimensional model was not a good fit for the data for any of the scoring methods, with none of the indices indicating acceptable fit. In particular, the RMSEA values of greater than 0.08 would normally lead to the rejection of this model [26]. Hence the simple one-dimensional model of the GHQ-12 was rejected.

### Model 2

Again, consistent with previous reports, Graetz' three-dimensional model was a good fit for the data, for all three scoring methods, on most indices. The RMSEA values of 0.073, 0.077 and 0.066 for the Likert method, GHQ method and C-GHQ method respectively suggest that the three-dimensional model was at least an adequate fit for the data, with the best fit being obtained from the C-GHQ scoring method. Hence the three-dimensional model was accepted.

### Model 3

The one-dimensional model incorporating response bias (Model 3) was the best fit overall for the data, with all fit indices indicating a better fit than the competing three-dimensional model. Consideration of the 90% confidence limits for the RMSEAs for the Likert method and C-GHQ method suggested substantially better fits than for the corresponding three-dimensional models, with the best fitting model overall being the C-GHQ scoring method (RMSEA = 0.053). Hence it was concluded that Model 3 was the best fit for the data.

### Reliability and discrimination of Model 3

Table 2 shows the reliability and discrimination estimates for Model 3. It can be seen that the conventional estimate of reliability provided by Cronbach's Alpha overestimated the reliability of the GHQ-12 by 3.3% to 39.7%, depending on the scoring method. This is because Cronbach's estimate is computed on the assumption that item errors are uncorrelated, a hypothesis that was rejected when Model 1 was rejected and Model 3 accepted. A more realistic estimate of reliability was given by the square of the implied correlation between the latent variable and the composite score (implied *r*^2^), and this suggested that the better estimate of reliability varied between 0.53 (C-GHQ method) and 0.874 (GHQ method). Subsequently the standard error of measurement (SEM) was 2.55 for the Likert method, 0.94 for the GHQ method and 2.01 for the C-GHQ method. Discrimination was also found to vary dramatically by scoring method, with Ferguson's Delta ranging from 0.626 (GHQ method) and 0.968 (C-GHQ method).

**Table 2:** Descriptive statistics, reliability and discrimination estimates for Model 3

***Scoring method***	***Scale mean***	***Scale SD***	***Alpha (α)***	***Implied r*^2^**	***Difference (α-implied r^2^)***	***SEM***	***Delta (δ)***
Likert	10.6	4.9	0.90	0.73	0.17	2.6	0.94
GHQ	1.4	2.7	0.90	0.87	0.03	0.9	0.63
C-GHQ	4.0	3.0	0.75	0.53	0.22	2.0	0.97

## Discussion

The best fitting model across all scoring methods was the one-dimensional model with response bias. The GHQ-12 appears to measure a single dimension, but with greater error on the negatively phrased items. In addition, the errors on the negatively phrased items appear to be correlated. While this confirms the previous report of the unidimensional nature of the GHQ-12, it suggests that the proposed mechanism of ambiguous response choices for the negatively phrased items is not fully responsible for the response bias. Indeed, the scoring methods that eliminate this ambiguity (GHQ/C-GHQ methods) still generate data more consistent with the response bias model than the alternative three-dimensional model. It has been suggested that, in general, response bias to negatively phrased items may be due to carelessness [11], e.g. the respondent fails to notice that the response format has changed, or education [12], e.g. difficulty reading statements containing negations. The latter may be ruled out since the GHQ-12 contains no negations, but the former might be investigated by adopting a uniform response format for all items.

While further research is required to isolate the exact cause of the response bias, the findings of this study confirm that the GHQ-12 is best thought of as a one-dimensional measure, and that previously reported multi-factor models are simply reporting artefacts of the method of analysis. The results also suggest, however, that the response bias can introduce a degree of error unacceptable for clinical psychometric measurement, which has not been previously recognised. Conventional estimates of reliability such as Cronbach's Alpha may under- or over-estimate reliability if the assumptions of classical test theory are not met. These assumptions should be examined and if necessary an alternative estimation method employed. In this study, Alpha was found to overestimate the reliability of the GHQ-12, since the estimate was based on the assumption of uncorrelated item errors. This has implications for the use of the GHQ-12 as a clinical psychometric measure: the reliability estimate of 0.534 for the C-GHQ scoring method was based on more realistic assumptions, and suggests that it should not be used to screen for psychiatric disorder because the resulting standard error of measurement of around two scale points creates a large degree of uncertainty around any threshold value. The GHQ scoring method produced the least measurement error (SEM = 0.94) but sacrificed discriminatory power in doing so, with Ferguson's Delta = 0.626 indicating that the scoring method failed to discriminate within 37.4% of the sample.

## Conclusion

Conventional psychometric assessments using factor analysis have led to the erroneous conclusion that the GHQ-12 is multidimensional, ignoring the possibility that this was an artefact of the analysis. Failing to take response bias into account has also obscured substantial measurement error in the GHQ-12, since conventional estimates of reliability assume that item errors are uncorrelated. Until the source of this response bias is identified and eliminated, the GHQ-12 would seem to have limited utility in clinical psychometrics, in particular as a screen for psychiatric morbidity.

## Competing interests

The author declares that they have no competing interests.

## Authors' contributions

MH is the sole author

## Pre-publication history

The pre-publication history for this paper can be accessed here:


